# Supplementary material for: Cognitive Functioning and Nail Salon Occupational Exposure among Vietnamese Immigrant Women in Northern California
Source: Int J Environ Res Public Health. 2022 Apr 12;19(8):4634. doi: 10.3390/ijerph19084634 (PMC9032223; doi:10.3390/ijerph19084634)
Supplement: Supplementary file 1 [file ijerph-19-04634-s001.zip › ijerph-1596235-supplementary.pdf]

Supplemental Table S1. Characteristics of Current full-time, Former, and Part-time Nail Salon Worker Participants.

	Current full-time Nail Salon Worker (n=136)	Former Nail Salon Worker (n=14)	Part-time Nail Salon Worker [a] (n=5)	Total (n = 155)	p-value [a]
	**N(%)**	**N(%)**	**N(%)**	**N(%)**	
**Education**					0.842
Elementary/middle school	37 (28.0%)	5 (35.7%)	2 (40.0%)	44 (29.1%)	
High school	55 (41.7%)	5 (35.7%)	1 (20.0%)	61 (40.4%)	
Technical school/Some college	30 (22.7%)	4 (28.6%)	2 (40.0%)	36 (23.8%)	
Bachelor/Master/Ph.D/MD/JD etc	10 (7.6%)	0 (0.0%)	0 (0.0%)	10 (6.6%)	
**Marriage status**					0.096
Single	8 (6.1%)	2 (14.3%)	0 (0.0%)	10 (6.6%)	
Married	82 (62.1%)	7 (50.0%)	1 (20.0%)	90 (59.6%)	
Divorced	19 (14.4%)	2 (14.3%)	1 (20.0%)	22 (14.6%)	
Widow	12 (9.1%)	3 (21.4%)	2 (40.0%)	17 (11.3%)	
Domestic partner	11 (8.3%)	0 (0.0%)	1 (20.0%)	12 (8.0%)	
**Ability to speak English**					0.526
Little	9 (6.8%)	0 (0.0%)	0 (0.0%)	9 (6.0%)	
Average	72 (54.55%)	10 (71.4%)	2 (40.0%)	84 (55.6%)	
Good/Great	51 (38.6%)	4 (28.6%)	3 (60.0%)	58 (38.4%)	
	**Mean (±SD)**	**Mean (±SD)**	**Mean (±SD)**	**Mean (±SD)**	
**Age**	57.1 (±5.9)	58.9 (±6.5)	59.4 (±7.3)	57.4 (±6.0)	0.462
**MoCA score**	17.7 (±4.4)	16.8 (±3.1)	19.0 (±5.8)	17.6 (±4.3)	0.627
**Education-adjusted MoCA score[b]**	18.0 (±4.3)	17.3 (±3.1)	19.6 (±5.2)	18.0 (±4.2)	0.603
**CES-D (Depression scale)**	16.3 (±9.9)	19.3 (±9.1)	17.0 (±8.8)	16.6 (±9.8)	0.370

a. P-values of comparing groups from Kruskal-Wallis tests for continuous and ordinal variables, and χ^2 test (or Fisher exact test) for categorical variables.

b. If participant did not finish high school, the education-adjusted MoCA score = MoCA+1; otherwise education-adjusted MoCA score = MoCA.

Supplemental Table S2. Summary of toxin-exposure of Current, Former, and Part-time Nail Salon Worker Participants. Raw means (standard deviations) and counts (proportions) are reported.

	**Current full-time Nail Salon Worker (n=136)**		**Former Nail Salon Worker (n=14)**		**Part-time Nail Salon Worker [a] (n=5)**		
	**Observed n**	**Mean (±SD) (Range) or N (%)**	**Observed n**	**Mean (±SD) (Range) or N (%)**	**Observed n**	**Mean (±SD) (Range) or N (%)**	**p-value [b]**
**Years working in current nail salon**	122	6.3 (±6.7) (0.04 - 27)	8	3.3 (±3.5) (0.5 - 11)	3	10.7 (±6.7) (3 - 15)	0.229
**Hours worked per week**	130	33.5 (±14.6) (4 - 70)	9	33.0 (±18.7) (1 - 70)	5	16.8 (±14.2) (3 - 40)	0.055
**Exposure Index [a]**	118	209.4 (±262.9) (1.25 – 1500)	4	100.0 (±120.0) (40 – 280)	3	229.0 (±321.3) (42 – 600)	0.769
**Environment in current workplace**							
Manitable	124	5.1 (±2.5) (0 - 12)	11	4.4 (±1.5) (1 - 6)	5	3.6 (±1.7) (1 - 5)	0.268
Pedichair	130	5.9 (±3.3) (0 - 15)	10	3.6 (±1.3) (1 - 5)	5	5.0 (±2.4) (3 - 9)	0.040
Lunch area	128	112 (87.5%)	12	9 (75.0%)	5	5 (100.0%)	0.370
Eating at the right time every day	104	43 (41.4%)	12	5 (41.7%)	5	1 (20.0%)	0.773
Front door	127	116 (91.3%)	12	11 (91.7%)	4	3 (75.0%)	0.378
Back door	126	46 (36.5%)	11	4 (36.4%)	5	2 (40.0%)	0.999
Table vent	115	69 (53.1%)	11	8 (6.2%)	4	3 (75.0%)	0.669
Table fan	123	111 (90.2%)	11	10 (90.9%)	5	4 (80.0%)	0.605

Ceiling fan	126	35 (27.8%)	11	4 (36.4%)	4	1 (25.0%)	0.792
Air condition	123	108 (87.8%)	9	8 (88.9%)	5	5 (100.0%)	0.999
Stationary vent	111	61 (55.0%)	10	5 (50.0%)	4	1 (25.0%)	0.553
Filtration	115	44 (38.3%)	10	5 (50.0%)	4	2 (50.0%)	0.588
**Self-rated quality of environment**							0.385
Horrible	131	2 (1.5%)	13	1 (7.7%)	5	0 (0.0%)	
Bad	131	11 (8.4%)	13	2 (15.4%)	5	0 (0.0%)	
Normal	131	55 (42.0%)	13	5 (38.5%)	5	1 (20.0%)	
Good	131	58 (44.3%)	13	4 (30.8%)	5	4 (80.0%)	
Great	131	5 (3.8%)	13	1 (7.7%)	5	0 (0.0%)	
**Work position**							0.863
Staff	132	111 (84.1%)	12	11 (91.7%)	5	5 (100.0%)	
Owner	132	21 (15.9%)	12	1 (8.3%)	5	0 (0.0%)	
**Service that current workplace offered**							
Pedi	130	130 (100.0%)	12	12 (100.0%)	4	4 (100.0%)	1.000
Mani	128	128 (100.0%)	12	12 (100.0%)	5	5 (100.0%)	1.000
Silk	119	54 (45.4%)	9	7 (77.8%)	5	1 (20.0%)	0.089
Gel	131	125 (95.4%)	12	11 (91.7%)	5	5 (100.0%)	0.582
Acrylics	130	118 (90.8%)	12	12 (100.0%)	5	5 (100.0%)	0.740
Extra	131	100 (76.3%)	12	7 (58.3%)	5	4 (80.0%)	0.319
**Service that participants offered**							
Pedi	131	127 (96.9%)	13	12 (92.3%)	5	5 (100.0%)	0.480
Mani	130	128 (96.2%)	13	12	5	5	0.547

				(92.3%)		(100.0%)	
Silk	121	30 (24.8%)	13	2 (15.4%)	5	0 (0.0%)	0.515
Gel	129	99 (76.7%)	13	4 (30.8%)	5	1 (20.0%)	<0.001
Acrylics	126	63 (50.0%)	13	5 (38.5%)	5	0 (0.0%)	0.073
Extra	115	47 (40.9%)	13	3 (23.1%)	4	1 (25.0%)	0.470
**Mask**							0.496
Never	132	28 (21.2%)	12	3 (25.0%)	5	2 (40.0%)	
Rarely	132	16 (11.4%)	12	0 (0.0%)	5	1 (20.0%)	
Often	132	43 (32.9%)	12	4 (33.3%)	5	2 (40.0%)	
Always	132	45 (33.6%)	12	5 (41.7%)	5	0 (0.0%)	
**Gloves**							0.257
Never	131	7 (5.3%)	12	2 (16.7%)	5	0 (0.0%)	
Rarely	131	4 (3.1%)	12	0 (0.0%)	5	0 (0.0%)	
Often	131	27 (20.6%)	12	3 (25.0%)	5	3 (60.0%)	
Always	131	93 (71.0%)	12	7 (58.3%)	5	2 (40.0%)	

a. Exposure index = (years working in current nail salon) x (hours worked per week).

Supplemental Table S3a. Sensitivity analysis on association between MOCA and factors using multiple linear regression in all participants, with Huber-White robust standard error estimator for potential heterogenous variances (n = 269).

	Estimate (95% CI)	p-value
**Education**		
Elementary/middle school	Reference	
High school	2.92 (1.73, 4.10)	**<0.001**
Technical school/Some college	4.61 (3.13, 6.09)	**<0.001**
Bachelor/Master/Ph.D/MD/JD etc	6.24 (4.57, 7.92)	**<0.001**
**Live alone**		
Yes	Reference	
No	0.02 (-0.98, 1.02)	0.975
**Ability to speak**		
Little	Reference	
Average	1.35 (-0.32, 3.03)	0.113
Good/Great	1.35 (-0.60, 3.30)	0.173
**Age (in years)**	-0.07 (-0.15, 0.02)	0.119
**Exposure index (in 100)[a]**	-0.29 (-0.49, -0.09)	**0.004**

a. Exposure index = (years working in current nail salon) x (hours worked per week). For non-nail workers, exposure index = 0.

Supplemental Table S3b. Sensitivity analysis on association between MOCA and factors using multiple linear regression in all participants, after multiple imputations [b] for missing variables (n = 300).

	Estimate (95% CI)	p-value
**Education**		
Elementary/middle school	Reference	
High school	2.91 (1.72, 4.10)	**<0.001**
Technical school/Some college	4.61 (3.19, 6.03)	**<0.001**
Bachelor/Master/Ph.D/MD/JD etc	6.25 (4.52, 7.98)	**<0.001**
**Live alone**		
Yes	Reference	
No	0.02 (-0.98, 1.02)	0.966
**Ability to speak**		
Little	Reference	
Average	1.33 (-0.27, 2.92)	0.104
Good/Great	1.35 (-0.45, 3.14)	0.142
**Age (in years)**	-0.06 (-0.14, 0.01)	0.097
**Exposure index (in 100)[a]**	-0.29 (-0.53, -0.05)	**0.018**

a. Exposure index = (years working in current nail salon) x (hours worked per week). For non-nail workers, exposure index = 0.

b. Fifty imputed values for each missing variable were generated using MICE (multiple imputation chained equations)

Supplemental Table S3c. Sensitivity analysis on association between MOCA and factors using multiple linear regression in nail salon workers only (n = 125).

	Estimate (95% CI)	p-value
**Education**		
Elementary/middle school	Reference	
High school	3.23 (1.47, 5.00)	**<0.001**
Technical school/Some college	5.75 (3.58, 7.92)	**<0.001**
Bachelor/Master/Ph.D/MD/JD etc	6.23 (3.19, 9.26)	**<0.001**
**Live alone**		
Yes	Reference	
No	0.16 (-1.46, 1.79)	0.842
**Ability to speak**		
Little	Reference	
Average	-0.55 (-3.42, 2.33)	0.709
Good/Great	-1.03 (-4.13, 2.06)	0.512
**Age (in years)**	-0.06 (-0.18, 0.07)	0.359
**Exposure index (in 100)[a]**	-0.19 (-0.48, 0.10)	0.204

a. Exposure index = (years working in current nail salon) x (hours worked per week).

Supplemental Table S3d. Sensitivity analysis on association between MOCA and factors using multiple linear regression in current full-time nail salon workers only (n = 118).

	Estimate (95% CI)	p-value
**Education**		
Elementary/middle school	Reference	
High school	3.28 (1.49, 5.07)	**<0.001**
Technical school/Some college	5.44 (3.22, 7.67)	**<0.001**
Bachelor/Master/Ph.D/MD/JD etc	6.21 (3.18, 9.23)	**<0.001**
**Live alone**		
Yes	Reference	
No	0.27 (-1.38, 1.93)	0.746
**Ability to speak**		
Little	Reference	
Average	-0.36 (-3.23, 2.50)	0.803
Good/Great	-1.14 (-4.23, 1.95)	0.469
**Age (in years)**	-0.09 (-0.22, 0.04)	0.180
**Exposure index (in 100)[a]**	-0.22 (-0.51, 0.08)	0.152

a. Exposure index = (years working in current nail salon) x (hours worked per week). For non-nail workers, exposure index = 0.
